# Oxygen uptake efficiency slope at anaerobic threshold can predict peak VO_2_ in adult congenital heart disease

**DOI:** 10.1016/j.ijcchd.2024.100546

**Published:** 2024-09-29

**Authors:** Thomas Simon FitzMaurice, Scott Hawkes, Yuen Liao, Damien Cullington, Angella Bryan, James Redfern, Reza Ashrafi

**Affiliations:** a Liverpool Centre for Cardiovascular Science, University of Liverpool, Liverpool, UK; b Liverpool Heart and Chest Hospital NHS Foundation Trust, Liverpool, UK; c University of Manchester, Manchester, UK; d Countess of Chester Hospital NHS Foundation Trust, Chester, UK

**Keywords:** Adult congenital heart disease, Cardiopulmonary exercise testing, Submaximal testing

## Abstract

**Introduction:**

Assessment of exercise capacity by cardiopulmonary exercise testing (CPET) in adults with congenital heart disease (CHD) is important for prognostication and preoperative assessment. Peak oxygen uptake (PVO_2_) is used commonly, but can be challenging due to the difficulties of undertaking maximal CPET testing in this population. We explored whether oxygen uptake efficiency slope (OUES) at ventilatory anaerobic threshold (VAT), the point during CPET at which OUES becomes strongly correlated with PVO_2_, and is more reliably available from submaximal CPET, can predict PVO_2_ in adults with CHD.

**Methods:**

We assessed consecutive individuals who completed maximal CPET at our cardiorespiratory centre, as part of routine service review, between March 2019 and August 2021, recording data such as PVO_2_, VAT and OUES at various proportions of a maximal test (75 %, 90 %, 100 %, and VAT). We employed linear regression modelling to analyse the association between PVO_2_ and OUES at VAT, and subsequently create an equation to predict PVO_2_ from OUES at VAT. Parametric data are presented using Pearson's correlation coefficient and non-parametric data using Spearman's rho.

**Results:**

We analysed 391 individuals (177 female, age 32 ± 11 years). Mean ± SD PVO_2_ was 23.3 ± 6.86 ml/min/kg or 1724 ± 540 ml/min, peak VE 86.7 ± 25.4 l/min. The point of VAT as a percentage of PVO_2_ achieved was 66.5 ± 9.4 %, and VAT as a percentage of predicted PVO_2_ 46.9 ± 11.4 %. PVO_2_ was correlated with OUES at 100 % (R = 0.91, P < .001), 90 % (R = 0.91, P < .001), 75 % (R = 0.89, P < .001) of maximum, and VAT (R = 0.83, P < .001). PVO_2_*(ml/min)* could be predicted by: *(OUES at VAT)∗685.245 + (BMI [kg/m*^*2*^*])∗5.045 + (FEV*_*1*_*[l])∗223.620 – 153.205*.

**Conclusions:**

OUES at VAT can be used to calculate PVO_2_. To our knowledge, this is the first equation using OUES at VAT to predict PVO_2_ in adults with CHD. In a population who may find maximal CPET difficult, this may be a useful submaximal measurement of cardiovascular fitness, and to calculate PVO_2_, which is commonly used in guideline-based decision making in CHD.

## 1 Introduction

Accurate calculation of exercise capacity and cardiopulmonary functional reserve in adults with congenital heart disease (CHD) is important due to the frequent need for surgical procedures [1], prediction of functional status [2,3], assessment of severity of heart disease, and prognostication [4] necessary in this population. Cardiopulmonary exercise testing (CPET) provides an objective assessment of an individual's exercise capacity. Peak oxygen uptake (PVO_2_) is calculated during CPET to determine an individual's aerobic fitness and is used commonly across a spectrum of health conditions, including adult CHD. PVO_2_ is defined as the highest rate of oxygen uptake that can be consumed, transported and utilised during a test to volitional exhaustion during incremental exercise [5]. PVO_2_ differs from VO_2_ max, the latter being defined as the maximum amount of O_2_ uptake beyond which no increase in rate of work will increase VO_2_, and the plateau that occurs in VO_2_ max may not be seen at PVO_2_ in many individuals undergoing CPET. However, both PVO_2_ and VO_2_ max can be challenging to obtain in this population as such individuals are often unable to reach maximal exercise due to factors such as poor motivation, abnormal heart rate response or musculoskeletal limitations [6]. Even if an individual is able to reach maximal exercise, a true plateau may not always be seen during CPET [3]; PVO_2_ is for these reasons used commonly and correlates well to population health in CHD, and has an extensive role in CHD outcomes, surgical and interventional research [[7], [8], [9]].

Various indices have been proposed to try to predict cardiopulmonary functional reserve from a submaximal exercise test. Ventilatory anaerobic threshold (VAT), or gas exchange threshold, is defined as the metabolic rate at which VCO_2_ increases disproportionately to VO_2_, due to an increase in the evolution of CO_2_ relative to the rate at which blood and muscle bicarbonate decrease as a result of buffering of a metabolic acidosis. VAT signals the point at which anaerobic metabolism exceeds aerobic metabolism, to such an extent that it greatly reduces exercise tolerance and time to exhaustion [10]. However, VAT may be contentious due to interobserver and intra-observer variability [11] and protocol dependence [12]. The minute ventilation (V_E_)/carbon dioxide production (V_CO2_) slope (V_E_-V_CO2_) [13] is used widely for prognostication in patients with chronic heart failure. Whilst V_E_-V_CO2_ is well correlated with New York Heart Association (NYHA) functional classification in ACHD patients [14], it correlates poorly with peak VO_2_ [15].

The oxygen uptake efficiency slope (OUES) was first described by Baba et al. in 1996 [16] in an attempt to create an objective and effort-independent estimate of cardiorespiratory functional reserve. OUES is the product of the linear relationship of oxygen uptake (VO_2_) versus the log of minute ventilation (V_E_) during exercise, expressed as VO_2_ = log10 (VE/VE rest) + VO_2_ rest [16]. PVO_2_ correlates well with OUES at 100 % of a maximal exercise test but if a submaximal test is performed, it can be difficult to calculate the percentage through a maximal test at which testing is stopped, limiting the real-world use of OUES as a submaximal measure of exercise capacity, since a defined percentage through the test needs to be known in order to calculate OUES at this point: below 50 % of a maximal test, the predictive power of OUES becomes weak [17]. Our group has demonstrated previously that PVO_2_ in an ACHD population correlated well with OUES at 100 %, 90 %, 75 %, and ventilatory anaerobic threshold (VAT) in a group of adults with CHD [18].

VAT is measured when the increase in VCO_2_ exceeds VO_2_, signalling the body's inability to meet oxygen consumption for the effort undertaken [19] and occurs commonly around halfway through CPET [20], and before PVO_2_ is reached [21]. VAT as a definition and its use clinically in CPET is discussed in depth elsewhere [22,23]. We have previously demonstrated good correlation between OUES and PVO_2_ at various percentage intervals of a maximal test in adults with CHD using CPET [18], and wished now to explore whether OUES at VAT, the point at which OUES becomes strongly correlated with PVO_2_ [18], could be used to reliably calculate PVO_2_ in a population of adults with CHD.

## 2 Materials and methods

As part of a routine service evaluation, we reviewed records of all patients aged 18 years and over with a confirmed diagnosis of adult CHD cared for under the Liverpool Heart and Chest Hospital NHS Foundation Trust Adult Congenital Heart Disease service (a large tertiary CHD service), who had undergone a CPET between March 2019 and August 2021. We excluded repeated tests on the same individual to avoid repeated measurement error, opting for the first maximal test performed. Individuals who were tachycardic (>100bpm) at rest, suffering an acute illness as defined by a clinician, or unable to provide informed consent for CPET did not have a CPET performed, therefore individuals meeting these criteria are not included in the data. Local approval as a service improvement project was gained from the Liverpool Heart and Chest Hospital NHS Foundation Trust.

### 2.1 Anthropometric data

Age, sex, CHD diagnosis, height in metres and weight in kilograms were recorded in all patients. Height was recorded using a stadiometer (Secca GmbH, Germany) and weight using electronic scales (Secca GmbH, Germany).

### 2.2 Lung function

Spirometry was performed routinely as part of the CPET protocol at our institution; this was performed at rest, to American Thoracic Society – European Respiratory Society (ATS-ERS) guidelines for acceptability and repeatability [24]. Spirometry was performed in a seated position using a Geratherm® Respiratory Ergostik™ spirometer (Geratherm®, Bad Kissingen, Germany) and results recorded using Geratherm® Blue Cherry™ software. Parameters recorded were forced expiratory volume in 1 s (FEV_1_) (in litres), forced vital capacity (FVC) (in litres) and FEV_1_/FVC ratio. Results were reviewed by a senior pulmonary physiologist (SH) of 12 years’ experience to ensure quality. Predicted values for spirometry used were derived from the Global Lung Index 2012 [25].

### 2.3 Cardiopulmonary exercise testing

CPET was performed using an electronically braked cycle ergometer (Ergoselect 100, Ergoline GmbH, Germany). CPET followed a standardised protocol [26]: 3 min at rest, 3 min unloaded cycling at 60 rotations per minute (rpm), followed by an incremental increase in work rate from 5 to 30 W/min, to elicit maximum performance within 8–12 min of exercise. Carbon dioxide production (VCO_2_ [ml/min]), VO_2_ (ml/min), ventilation (VE [l/min]) and respiratory rate were measured using breath-by-breath gas analysis through a face mask connected to a metabolic cart (Geratherm GmbH, Germany). The test was stopped when patients achieved volitional exhaustion and were either unable to maintain the required cadence of 60–80 rpm or all the identifiers of a maximal exercise test were present (RER ≥1.1, and a sustained plateau of VO_2_). The widespread use of cardiac pacemakers and rate-limiting medication in our population precluded the use of peak heart rate as a marker of a maximal test. Those who did not achieve a maximal test (premature cessation of test prior to exhaustion, for example due to cardiac dysrhythmia, hyperventilation or patient choice) were excluded from recording. PVO_2_ was determined as the highest 30 s average of VO_2_. VAT was calculated using the V-slope method and VE/VCO_2_ was determined at VAT [16]. CPET resting, predicted and peak data was recorded using predicted equations (full details of these equations can be found as a supplement) [27].

### 2.4 Oxygen uptake efficiency slope

We calculated OUES, the product of the linear relationship of oxygen uptake (VO_2_) versus the log of minute ventilation (V_E_) during exercise, using the formula VO_2_ = log10 (VE/VE rest) + VO_2_ rest [16]. We calculated OUES breath by breath, including the last minute of rest, following a period of mask familiarisation, and recorded OUES at specific intervals (VAT, 75 %, 90 % and 100 % of a maximal test (determined as the highest 30 s average of oxygen uptake). VAT was calculated using the V-slope method. OUES at specific intervals was calculated by removing all data beyond that specific interval of total exercise time and recalculating OUES from the remaining data. Raw data were used to make these calculations.

### 2.5 Statistical analysis

Statistical analysis was conducted using Rstudio, v.1.2.5 (Rstudio, Vienna, Austria). The distribution of variables was assessed using the Shapiro-Wilk test and visualised using quantile-quantile plots. Preliminary analyses were conducted to ensure no violation of the assumptions of linearity, normality, homoscedasticity and multicollinearity. Normally distributed data are reported where appropriate as mean ± standard deviation (SD), and non-normally distributed data using median (interquartile range). Correlations were assessed using the Pearson product-moment correlation coefficient if the data were parametric and using Spearman's rho if non-parametric. A step wise linear regression modelling strategy was employed using the ‘caret’ package for R to analyse the association between PVO_2_ and OUES at VAT. Along with OUES at VAT, age, height, weight, body mass index, FVC and FEV_1_ were explored as explanatory variables in the model selection strategy. Interdependent variables such as height and BMI were not assessed concurrently to avoid collinearity in the model. Model testing was performed using 10-fold cross-validation and reported using root mean square error (RMSE) and mean average error (MAE) [28]. Agreement between PVO_2_ calculated by CPET and modelled PVO_2_ was visualised using a Bland-Altman plot [29]. Comparisons of differences between groups were made using Student's paired T-test. P values of < 0.05 were considered significant.

## 3 Results

Of the 435 consecutive individuals undergoing CPET, we excluded 41 as CPET was performed to a submaximal level, and a further 3 for incomplete data recording such as missing anthropometric or spirometric information in electronic patient records, leaving 391 eligible individuals (177 female, age 32 ± 11 years). Full anthropometric characteristics are described in Table 1; lesion characteristics are described in Table 2. Average PVO_2_ was 23.3 ± 6.86 ml/min/kg or 1724 ± 540 ml/min and average peak VE was 86.7 ± 25.4 l/min. The average point of VAT as expressed as a percentage of the PVO_2_ achieved was 66.5 ± 9.4 %, and VAT as a percentage of the predicted PVO_2_ was 46.9 ± 11.4 %.

**Table 1:** Demographic information and average CPET results.

**Variable**	**Number**
Age (years)	32 ± 11
Sex	177 female (45.3 %)/214 male (54.7 %)
Height (cm)	169 ± 10
Weight (kg)	76 ± 19
Body mass index (BMI) (kg/m^2^)	26 ± 6
FEV_1_ (percent predicted)	85 ± 17
FVC (percent predicted)	86 ± 16
FEV_1_/FVC ratio	81 ± 9
Restrictive pattern on spirometry^a^	76 (19.4 %)
Obstructive pattern on spirometry^a^	33 (8.4 %)
Bethesda mild lesion	43 (11 %)
Bethesda moderate lesion	178 (45.5 %)
Bethesda severe lesion	170 (43.5 %)
Peak VO_2_ (ml/min)	1724 ± 540
Peak VO_2_ (ml/min/kg)	23.3 ± 6.86
% of predicted peak VO_2_ achieved	71 ± 15
Peak VE (l/min)	86.7 ± 25.4
VAT as a percentage of predicted VO_2_	46.9 ± 11.4
VAT as percentage of achieved VO_2_	66.5 ± 9.4
VE/VCO_2_ at VAT	30.6 ± 5.5
Peak RER	1.3 ± 0.1
OUES as percent predicted	62 ± 14

a Based on the GLI lower limits of normal [25].

**Table 2:** Description of primary lesion.

**Primary lesion**	**Number**	**Percentage**
Isolated aortic valve disease	34	8.7
Isolated mitral valve disease	5	1.3
Atrial septal defect	14	3.6
Ventricular septal defect	8	2.0
Atrioventricular septal defect	13	3.3
Coarctation of the aorta	11	2.8
Sub-aortic stenosis	14	3.6
Anomalous venous drainage	5	1.3
Right ventricular outflow obstruction	19	4.9
Tetralogy of Fallot	82	21
Ebstein's anomaly	15	3.8
Pulmonary atresia	17	4.3
Transposition of the great arteries – systemic left ventricle	34	8.7
Transposition of the great arteries – systemic right ventricle	40	10.2
Congenitally corrected transposition of the great arteries	14	3.6
Fontan circulations	62	15.9
Other	4	1.0

**Fig. 1:**
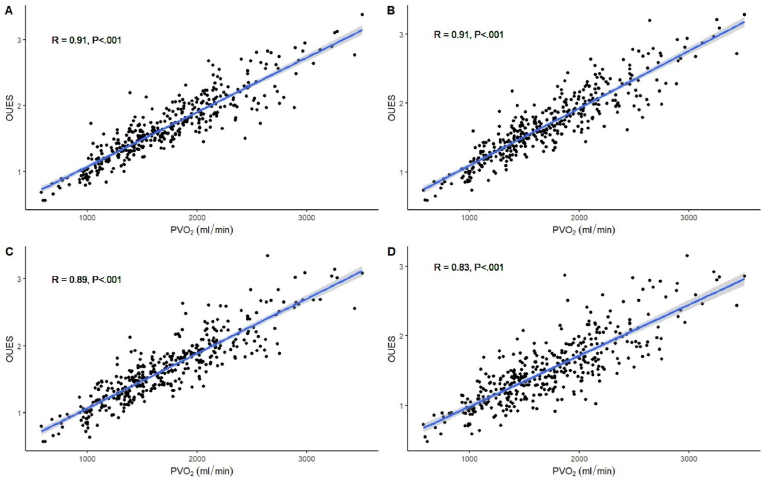
– Correlations between PVO_2_ and OUES at different intervals of a maximal test. Correlation between PVO_2_ and OUES at (A) 100 %, (B) 90 %, (C) 75 % of a maximal test and (D) at VAT.

PVO_2_ was strongly positively correlated with OUES at 100 % (R = 0.91, P < .001), at 90 % (R = 0.91, P < .001), at 75 % (R = 0.89, P < .001) of maximum, and at VAT (R = 0.83, P < .001). Correlations between OUES at 100 %, 90 % and 75 % of maximum and at VAT with PVO_2_ are shown in Fig. 1.

We used linear regression modelling to construct an equation to predict PVO_2_:

Peak VO_2_ (ml/min) = (OUES at VAT)∗685.245 + (body mass index [kg/m^2^])∗5.045 + (FEV_1_ [l])∗223.620–153.205.

**Fig. 2:**
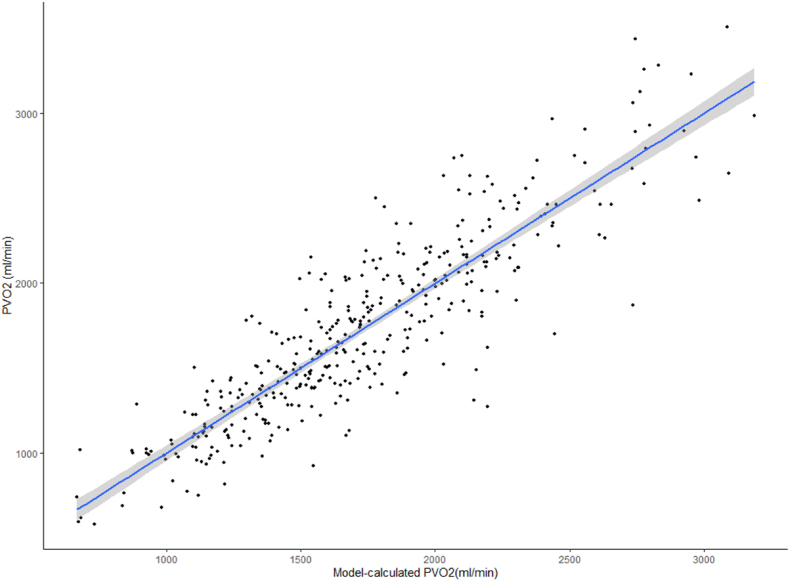
Correlation between PVO_2_ and OUES-calculated PVO_2_. Peak VO_2_ (ml/min) can be calculated by the formula: (OUES at VAT)∗685.245 + (body mass index [kg/m^2^])∗5.045 + (FEV_1_ [l])∗223.620–153.205.

**Fig. 3:**
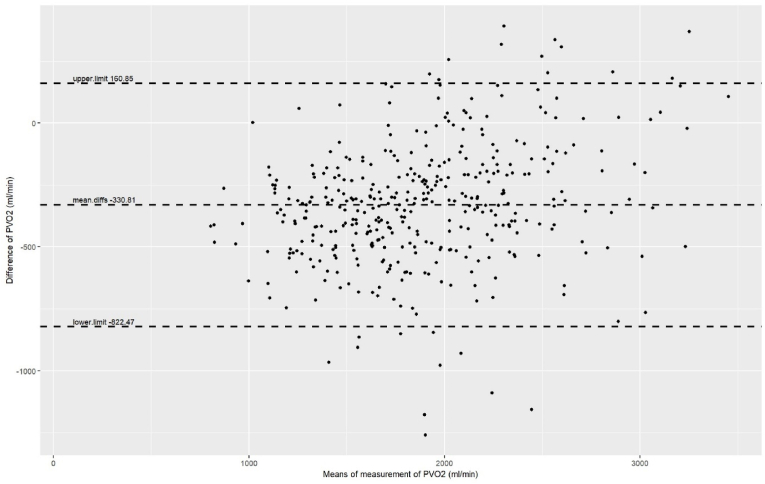
– Bland-Altman plot of PVO_2_ model.

The RMSE for the model was 256 ml/min, MAE 194 ml/min, and the adjusted R^2^ 0.78. There was no significant difference between the PVO_2_ values achieved or those calculated by the model (P = .99). Correlation between PVO_2_ calculated from the equation against actual PVO_2_ is shown in Fig. 2, and agreement in Fig. 3. OUES at VAT made the largest unique contribution to explaining the variance in the model (partial coefficient 23 %) with BMI and FEV_1_ 0.3 % and 9.7 % respectively. Bias was calculated at −331 (95 % confidence intervals −306 to −356, upper limits of agreement 161, lower limit of agreement −822).

## 4 Discussion

We have demonstrated that in a group of 391 adults with CHD, PVO_2_ was highly correlated with OUES at 100 % (R = 0.91, P < .001), at 90 % (R = 0.91, P < .001), at 75 % (R = 0.89, P < .001) of maximum and at VAT (R = 0.83, P < .001). In our population, we found VAT to fall within 66.5 ± 9.4 % a maximal CPET Using linear regression modelling, we determined that OUES at VAT can be used to calculate PVO_2_ using the equation: PVO_2_ (ml/min) = (OUES at VAT)∗685.245 + (Body mass index)∗5.045 + (FEV_1_)∗223.620–153.205. To our knowledge, this is the first report of an equation using OUES at VAT to predict PVO_2_ in adults with CHD. In a population who may not always be able to perform a maximal test, this may be a useful submaximal measurement of cardiovascular fitness. As PVO_2_ is a well utilised marker that is used to guide follow up [30] and potentially to plan treatments in CHD[31], the ability to have alternate ways to generate this value may be beneficial where patient ability, compliance or time resources maybe be a factor.

OUES is a well-established measure of cardiovascular fitness known to correlate with PVO_2_ [16,17,32,33] and we have demonstrated previously its applicability to this population [18]. Our reports of the correlation between OUES at various defined points of a maximal test are consistent with our own earlier data [18] and other reports in the literature [17,33]. However, some studies in other conditions have demonstrated weaker associations between OUES and PVO_2_ [34], and caution should be used when extrapolating our data to other populations.

We included all eligible service users undergoing CPET at our service, and a large range of congenital abnormalities, including Fontan, Tetralogy of Fallot and transposition of the great arteries, making the generalisability of our findings to the wider CHD population likely.

In this work, we chose VAT as a point of calculation to generate a predicted PVO_2_ as this seemed a reasonable point that could be identified early in a CPET (assuming this point is reached before volitional exhaustion occurs), and can be measured even in a deliberately submaximal study [35] or in standard testing [36]. VAT (or VO_2_ at lactate threshold) is not volitionally influenced by the patient and is well-documented in clinical CPET, albeit as a submaximal response. We acknowledge that the identification of defined point can be influenced by intra-operator variability, but with the modified V-slope method there are backup criteria in examining hyperventilation relative to oxygen and CO_2_ at that point. This may be an important limitation when considering VE VCO_2_ at VAT compared to the VEVCO_2_ slope as it relies on a single datum, however the OUES method described here utilises the whole slope, thus any inaccuracy in the defined endpoint will be small when considering 200–300 breath-by-breath data points. In addition, the prescribed method is using a slope to a well-defined and non-volition controlled end-point and as such is preferable to a peak comparison which may be very different across the cohort. Therefore, as VAT is calculated by an agreed and reproducible method here, we feel the variability should be low, and previous data suggests good correlation if this approach is taken [37]. Despite this, it should be pointed out that VAT may be difficult to ascertain in up to 10 % of individuals with severe heart failure [38], which must be taken into consideration when applying our formula. OUES as a function of oxygen consumption and minute ventilation may offer complimentary data from a physiological perspective to PVO_2_, as it may reflect ventilatory efficiency relative to oxygen consumption in a more complete way [39]. Additionally, given the association with metabolic acidosis and CO_2_ clearance to ventilatory drive it may be more useful in the CHD cohort, where there are complex exercise-related physiological relationships [40] and where additional information about ventilatory function maybe useful [41]. OUES as a marker similar to PVO_2_ may be useful if measured sequentially in response to exercise, as it can be improved seemingly without changes in central haemodynamics [39].The linear relationship of OUES and the ability to calculate a PVO_2_ for which there is more historical clinical data in the CHD cohort or citation in guidelines [42] may allow its wider incorporation or usage in the future, especially as it reflects both systemic and pulmonary function.

Whilst this work was designed to be able to calculate the PVO_2_ prospectively from submaximal tests as PVO_2_ is still amongst the most commonly used criteria in complex decision making in cardiology [43], we believe that there is extra utility in having a marker that may add more than PVO_2_ in isolation. We believe that OUES may offer extra utility in submaximal testing now that it can be shown to be used to calculate PVO_2_, probably one of the most commonly-referenced markers in CPET, clinicians may be able to feel more comfortable with an OUES valve in tandem with PVO_2_. OUES offers several uses: it is a marker of oxygen uptake efficiency [16], provides prognostic data [44], is related to skeletal muscle function [45], can be measured sub-maximally in a CPET [33] and can hep differentiate between respiratory and cardiac dyspnoea [46].

Our study was single-centre, and we did not include any non-CHD healthy comparators. Validation in a larger group is desirable, to ensure the applicability of our equation in a wider population, with closer attention paid to the proportions of different congenital cardiac abnormalities representative of the wider population. We excluded individuals who were unable to complete a maximal exercise test. This may introduce a degree of selection bias, since the very individuals in whom derivation of PVO_2_ from a submaximal test is necessary, might differ from those included in this study, who were necessarily able to complete a maximal test. The large amount of historical data on submaximal OUES calculation and the linear behaviour of OUES through exercise [47] does make us believe that the data and calculation above would not be significantly affected. To counter this, it is unlikely that inclusion of individuals unable to complete a maximal test would be possible since a maximal test was needed as a reference for calculation of submaximal OUES equations. Similarly, the use of secondary criteria may not be a reliable indicator of a maximal test and must be interpreted with caution [[48], [49], [50]]. Our study did not distinguish between cyanotic and non-cyanotic abnormalities, which might have been a meaningful factor in predictive modelling, though our previous work has shown that such subgroups may perform closely enough for the generalisability of our equation to be useful in routine practice [18]. Future studies may wish to investigate this further, and whether VAT falls at the same point, thus producing the same OUES value, in the same individuals over repeated measurements, and to test the repeatability of our predictive equation. We would consider the RMSE (256 ml/min) and MAE (194 ml/min) are small – variability in PVO_2_ can be expected [51], and may occur due to testing equipment [52], position [53] or over time [54] – but further work is desirable to assess whether the RMSE and MAE of our model are acceptable in its clinical applications, for example in assessment of thresholds for surgical fitness.

## 5 Conclusion

In conclusion, we have demonstrated that by using OUES at VAT our equation can be used to calculate PVO_2_ in adults with CHD. We believe that these data will offer clinicians the utility of OUES, but also PVO_2_ – a well-validated and established marker that many will be more familiar with – from a submaximal test. We believe that this can then be tested in a wider cohort of patients including non-CHD, such as perioperative assessment, patients for vascular surgery with limited mobility or patients with significant thoracic conditions where a full CPET may not be possible.

## CRediT authorship contribution statement

**Thomas Simon FitzMaurice:** Writing – review & editing, Writing – original draft, Project administration, Methodology, Data curation. **Scott Hawkes:** Resources, Methodology, Investigation, Data curation, Conceptualization. **Yuen Liao:** Data curation. **Damien Cullington:** Supervision. **Angella Bryan:** Methodology, Formal analysis. **James Redfern:** Supervision, Conceptualization. **Reza Ashrafi:** Writing – review & editing, Supervision, Conceptualization.

## Declaration of competing interest

The authors declare that they have no known competing financial interests or personal relationships that could have appeared to influence the work reported in this paper.
