# Supplementary material for: Oxygen uptake efficiency slope at anaerobic threshold can predict peak VO2 in adult congenital heart disease
Source: Int J Cardiol Congenit Heart Dis. 2024 Sep 29;18:100546. doi: 10.1016/j.ijcchd.2024.100546 (PMC11658284; doi:10.1016/j.ijcchd.2024.100546)
Supplement: Multimedia component 1 [file mmc1.docx]

# Supplement

Predicted peak VO_2­_ in ml/min:

1. Sedentary males

Cycle factor = 50.72 - 0.372 X A

Normal (predicted) W = 0.79 x (H - 60.7)

Actual W = normal W: Predicted PVO_2_ = Actual W x cycle factor
Actual W < normal W: Predicted PVO_2_ = (Normal W+ Actual WW2] x cycle factor.
Actual W > normal W: Predicted PVO_2_ = (Normal W x Cycle factor) + 6 x (Actual W- Normal W)

2. Sedentary females

Cycle factor = 22.78 – 0.17 xA.

Normal (predicted) W= 0.65 X H- 42.8.

Actual W = normal W: Predicted PVO_2_ = (Actual W+ 43) x Cycle factor.
Actual W < normal W: Predicted PVO_2_ = [(Normal W+ Actual W+ 86)/2] x Cycle factor.
Actual W > normal W: Predicted PVO_2_ = [(Normal W+ 43) × Cycle factor] +6X (Actual W - Normal W)

For all groups: if a treadmill is used rather than a cycle: multiply predicted peak VO_2_ by 1.11

Where W = weight in kg, H = height in cm, A = age in years

*Adapted from Hansen JE, Sue DY, Wasserman K. Predicted values for clinical exercise testing. Am Rev Respir Dis 1984;129(Suppl):S49-S55 and Bruce RA, Kusumi F, Hosmer D. Maximal oxygen intake and nomographic assessment of functional aerobic impairment in cardiovascular disease. Am Heart J 1973; 85:546-562.*
